# Puerperal Fever

**Published:** 1900-08-18

**Authors:** 


### PUERPERAL FEVER.

The obstetrical sections of the congresses both in Paris and in Ipswich not unnaturally busied themselves in discussing the most frequent cause of childbed mortality, namely, puerperal septicæmia. Dr. Smyly, in a more or less historical review of the subject, described the gradual growth of our knowledge as to the cause of puerperal fever, and urged upon his hearers various points which are now sufficiently well recognised in theory, although probably much neglected in ordinary practice, as those upon which the safety of the mother principally depended. His remarks upon the use of the routine douche are not without interest. Describing how, on the introduction of antiseptics into general surgery, it seemed but logical that they should be employed to remove all possible septicity from the vagina of the puerperal woman, he said that a time came when not only was the vagina douched before and after labour, but the uterus was included in the process, and that, finally, prophylactic douching reached its climax when permanent irrigation was introduced by Schücking. Soon, however, accidents and even deaths, the result of the douche, began to multiply, and it became clear that the douching was a cause of the very disease it was intended to prevent. The question—for it was still a question, there being distinguished obstetricians who still resort to prophylactic douching — resolved itself, he said, into the probability, or even the possibility of auto-infection, and he described the various experiments which went to show that not only were the discharges of the vagina inimical to septic organisms, but that removal of these discharges, even by strong antiseptics, left the vagina in a less protected position than before. From personal experience he felt certain that routine douching had been productive of much more harm than good. So far as antiseptics were concerned it was on the hands of the attendant that attention must be concentrated, and he thought he would not be far from the truth in saying that if there were no handling there would be no septic infection. It had to be remembered that by no known process could the hands be rendered absolutely aseptic, and thus three points must be insisted on, namely: To avoid as far as possible all septic contamination; to spare no pains to render the hands as innocuous as possible; and to restrict local interference within the narrowest limits; the last of these points including not only unnecessary operative interference, but also all needless vaginal examination. Next to antiseptics Dr. Smyly considered the substitution of external for internal examination manipulations as the most important advance in modern midwifery. By external manipulation, he said, we can tell whether the woman is pregnant or not, and the period to which the pregnancy has advanced; the presentation and position of the fœtus; whether it be living, dying, or dead; and whether there be one or more children in the uterus. We can also tell whether she is actually in labour or not, and the course and progress of the labour may be followed with accuracy. The external examination is then the one chiefly to be depended upon, not only because it is safer, but because it is easier and more reliable, and because it gives more information to the examiner. "Having for many years practised the vaginal examination exclusively, I found it difficult," he said, "to persuade myself of the truth of the latter assertion; but not only has my own individual experience proved to me that this is so, but in the Rotunda Hospital, where the students are obliged to write down upon the bed-cards the result of each examination, I found that mistakes in diagnosis were much more frequent with the vaginal than with the abdominal method." There are two things, however, that can only be made out by vaginal examination, namely, the condition of the os uteri, and a prolapse of the cord. But if such an examination has to be made the practitioner should remember that it is an operation never entirely devoid of risk, and whatever the method employed for the disinfection of the hands, it should be carried out with the greatest thoroughness. If it is true, or even only partially true, as has been urged by Hegar, Boxall, and Cullingworth, that notwithstanding the advances made in hospital work there is as yet no improvement in the mortality from septic disease in private midwifery practice, it proves, said Dr. Smyly, that those who undertake the care of parturient women are not yet alive to the responsibilities of their calling. Turning, however, to the proceedings in Paris, it would seem that, although on the whole the same conclusions were accepted as were expressed by Dr. Smyly, there was a somewhat greater tendency to admit the possibility of the occurrence of an autogenetic infection, especially in cases in which portions of the placenta might have been retained, the infection resulting from certain anaërobic organisms which had become saprophytic in the vaginal secretion. Speaking on the question of therapeutic treatment when unfortunately septicæmia has by some means or another been set up, M. Draghiescu, of Bucharest, described the local treatment which had been employed for some years in the maternity of Bucharest in cases of puerperal septicæmia. This consisted in plugging the uterine cavity with iodoform gauze soaked in a five per cent. or a ten per cent. solution of carbolic acid, after a preliminary prolonged intra-uterine irrigation. To have much chance of success this treatment should be undertaken as soon as the diagnosis is made. In the service at his hospital they had a fixed rule, that as soon as a newly confined woman had several shivers, a temperature of 38 deg. Cent., and a pulse of 100 a minute, especially if the case had been grave from the commencement, one ought to make a prolonged irrigation, followed by tamponment of the cavity of the uterus. He pointed out that other practitioners had used iodoform gauze in the uterine cavity, but rather as a means of drainage; but that his proceeding, which involved not only the draining but the distension of the cavity to a certain degree, had for its aim the cauterisation of the superficial layer of the uterine mucous lining. The tampon should be renewed twice in 24 hours, but how many times the proceeding was to be repeated the author of the paper did not say.

